# Genetic screening for anticancer genes highlights *FBLN5* as a synthetic lethal partner of *MYC*

**DOI:** 10.1186/s12964-023-01300-3

**Published:** 2023-10-20

**Authors:** Motasim Masood, Qize Ding, Adam D. Cawte, David S. Rueda, Stefan W. Grimm, Ernesto Yagüe, Mona El-Bahrawy

**Affiliations:** 1 Faculty of Medicine, Imperial College London, Du Cane Rd, London, UK; 2 Department of Medicine, Faculty of Medicine, Imperial College London, Du Cane Rd, London, UK; 3 Single Molecule Imaging Group, MRC London Institute of Medical Sciences, Du Cane Rd, London, UK; 4 Department of Infectious Disease, Faculty of Medicine, Imperial College London, Du Cane Rd, London, UK; 5 Department of Surgery and Cancer, Faculty of Medicine, Imperial College London, Du Cane Rd, London, UK; 6 Department of Metabolism, Digestion and Reproduction, Faculty of Medicine, Imperial College London, Du Cane Rd, London, UK; 7 Department of Pathology, Faculty of Medicine, University of Alexandria, Alexandria, Egypt

**Keywords:** Apoptosis, Cell death assay, Targeted therapy, Comparison pathway analyses, Causal analysis, TRAIL, Non-coding RNA, microRNA, Live cell imaging

## Abstract

**Background:**

When ectopically overexpressed, anticancer genes, such as *TRAIL*, *PAR4* and *ORCTL3*, specifically destroy tumour cells without harming untransformed cells. Anticancer genes can not only serve as powerful tumour specific therapy tools but studying their mode of action can reveal mechanisms underlying the neoplastic transformation, sustenance and spread.

**Methods:**

Anticancer gene discovery is normally accidental. Here we describe a systematic, gain of function, forward genetic screen in mammalian cells to isolate novel anticancer genes of human origin. Continuing with over 30,000 transcripts from our previous study, 377 cell death inducing genes were subjected to screening. *FBLN5* was chosen, as a proof of principle, for mechanistic gene expression profiling, comparison pathways analyses and functional studies.

**Results:**

Sixteen novel anticancer genes were isolated; these included non-coding RNAs, protein-coding genes and novel transcripts, such as *ZNF436*-AS1, *SMLR1*, *TMEFF2*, *LINC01529*, *HYAL2*, *NEIL2*, *FBLN5*, *YPEL4* and *PHKA2*-processed transcript. *FBLN5* selectively caused inhibition of *MYC* in COS-7 (transformed) cells but not in CV-1 (normal) cells. *MYC* was identified as synthetic lethality partner of *FBLN5* where *MYC* transformed CV-1 cells experienced cell death upon *FBLN5* transfection, whereas *FBLN5* lost cell death induction in MCF-7 cells upon *MYC* knockdown.

**Conclusions:**

Sixteen novel anticancer genes are present in human genome including *FBLN5*. *MYC* is a synthetic lethality partner of *FBLN5*.

Video Abstract

**Supplementary Information:**

The online version contains supplementary material available at 10.1186/s12964-023-01300-3.

## Background

Despite leaps forward in our understanding of cancer biology, comparatively little progress has been made when it comes to treatment. The slight improvement in the mortality rates of some cancers can be largely attributed to the progress made in cancer diagnostics and prevention [1]. For chemotherapeutic agents, the specificity against cancer cells is primarily quantitative rather than qualitative, with the obvious drawback of generalized cytotoxicity, consequently leading to an increase in patient morbidity and mortality [2]. Despite the earlier success of targeted therapies, recent data suggest that among an unselected population of patients, the response rate of targeted therapies is usually limited to 10–20% with clinical response typically lasting 6–12 months [3].

Anticancer genes, a new class of recently identified genes, specifically destroy tumour cells upon ectopic overexpression, such as TRAIL [4, 5]. Of the ten anticancer genes reported to date, some have viral origins but others are found in mammalian genomes, such as *TRAIL*, *MDA7*, *PAR4* and *ORCTL3* [5]. The tumour specific function of anticancer genes is based on the principle of synthetic lethality: a type of genetic interaction where the co-existence of two genetic alterations results in cell or organismal death and was first discovered in *Drosophila melanogaster* [5]. Whilst conventional chemotherapeutics block the activities of proteins and pathways essential for cancer cell growth and proliferation, upon ectopic overexpression anticancer genes can actively initiate signals in a dominant way that generate conflicts with malignant transformation signals, ultimately leading to transformed-cell specific cell death. In contrast, tumour suppressor genes act passively by preventing tumour formation at their endogenous expression levels. The mode of action of anticancer genes is not limited to activation, i.e. Orctl3 functions as a cation transporter in normal cells but when overexpressed in HRAS-transformed CV-1 cells, it inhibits stearoyl-CoA desaturase (*SCD*) and disrupts the fatty acid metabolism pathway crucial for rapidly dividing cells [6].

Anticancer genes have opened a new avenue of potential therapeutic intervention, and some, such as *TRAIL, MDA7* and HAMLET, have reached phase I/II clinical trials, whereas others are still in the preclinical stage of research [4]. In addition to tumour specific synthetic lethality, anticancer genes offer additional distinct advantages over conventional therapy, e.g., apoptin can initiate multiple signalling pathways in the cell [7], which multiplies its potential of creating synthetic lethal conflicts. Instead of solely relying on apoptosis, anticancer genes can impart alternate sub-modalities of regulated cell death like necrotic cell death and lysosome-dependent cell death [4], thus providing added benefits as the apoptosis machinery is usually defective in cancer cells. In addition, the inherent complexity of protein products of anticancer genes makes them more specific than low molecular weight compounds, making it possible therapeutic intervention of pharmacologically undruggable targets such as MYC, mutated RAS, RB and p53 [8, 9].

**Fig. 1:**
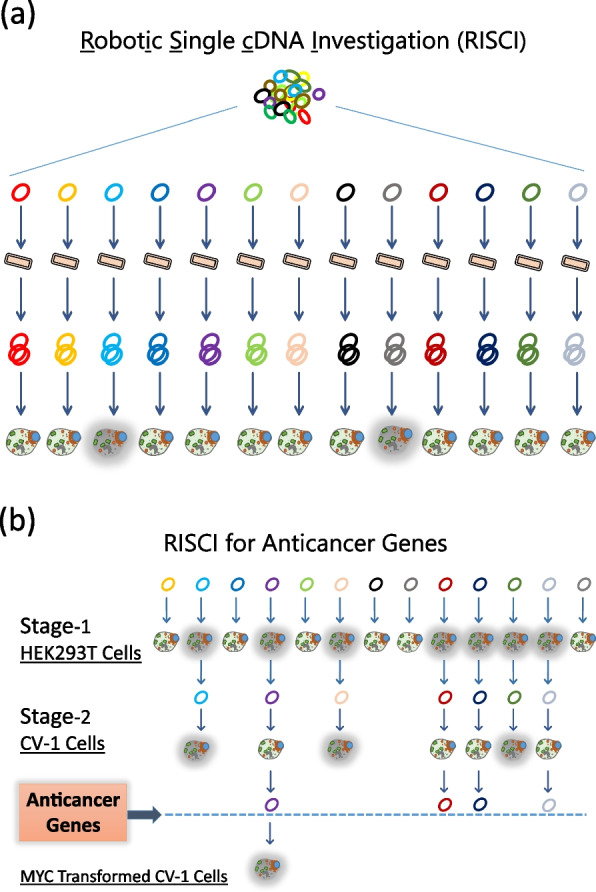
Schematic representation of the screening for anticancer genes through robotic single cDNA investigation (RISCI) process. **a** Employing robotic automation, plasmids are propagated in *E. coli*, DNA isolated and transfected in mammalian cells grown in 96-well plates with one gene per well and assayed for the desired phenotype (grey mask) — frequently cell death. **b** Cell death inducing genes in HEK293T cells were isolated through RISCI. These genes were transfected into untransformed CV-1 cells and a subset, which did not cause cell death, was identified as anticancer genes. One of the anticancer genes identified, *FBLN5*, was also transfected into *MYC*-transformed CV-1 cells resulting in cell death

We have previously reported the discovery and characterization of anticancer gene *ORCTL3* (an orphan transporter protein) using a novel robotic single cDNA investigation methodology, RISCI [10] (Fig. 1a), and later reported that *ORCTL3* triggers apoptosis through inhibition of *SCD* in HRAS-transformed CV-1 cells, an adult African green monkey (*Chlorocebus aethiops*) kidney cell line [6]. Here, using a small-scale gain of function forward genetic screen, we are reporting the isolation of 16 novel anticancer genes based on their ability to cause cell death in HEK293T, HeLa and MCF-7 cells and not in the CV-1 cells. From this screen Fibulin-5 (*FBLN5*) was selected as the proof of principle for further analysis (Fig. 1b). Using CV-1 cells as a model for normal cells and COS-7 cells as a model for transformed cells (SV40-transformed CV-1 cells) [11] we confirmed *FBLN5* induces cell death in COS-7, but not in CV-1 cells. Through gene expression profiling, comparison-ontology analyses and subsequent functional studies, *FBLN5* was confirmed as a synthetic lethality partner of *MYC*, causing selective downregulation of *MYC*-dependent genes in COS-7 cells and inducing *MYC*-specific cell death in COS-7 and MCF-7 breast cancer cells.

## Methods

### Plasmids

**Fig. 2:**
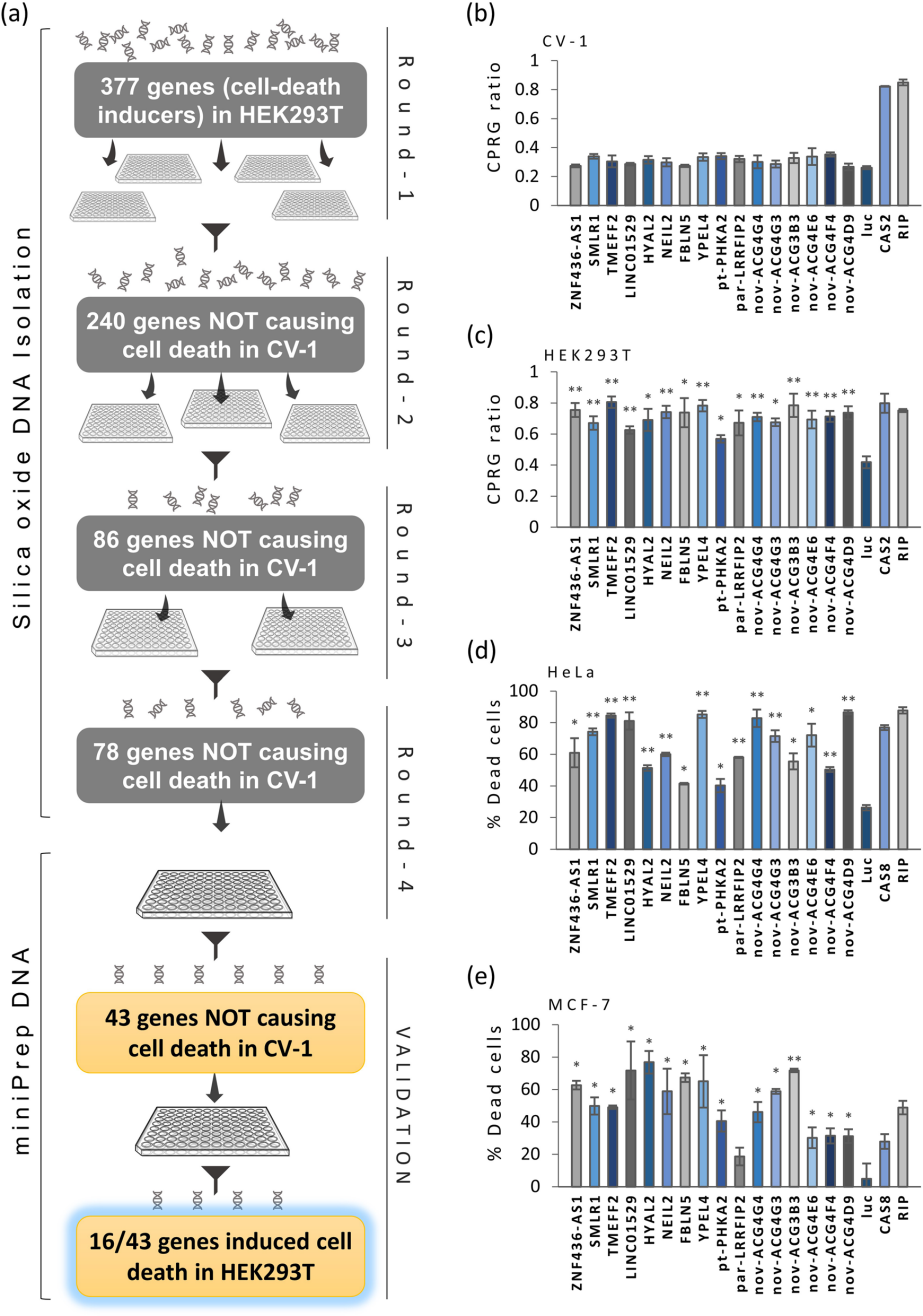
Sixteen novel anticancer genes cause cell death in transformed cells but not in normal cells. **a** Diagram depicting the process undertaken for the isolation of 16 novel anticancer genes. Screening was performed in two steps: in the first step a subset of 377 cell death inducing genes (in HEK-293 T cells) was transfected into non-transformed CV-1 cells in 4 consecutive rounds of screening where genes causing cell death above the internal threshold set for each round were gradually eliminated, resulting in a set of 78 gene candidates. DNA was isolated using silica oxide purification. In the second step, the plasmids were purified using standard DNA-miniPrep and another 35 genes were eliminated in CV-1 cells. The resulting 43 genes were transfected into HEK293T cells and 16 genes were selected for their ability to cause cell death in HEK-293T cells but not in CV-1 cells. **b**, **c** Relative cell death (CPRG ratio) in CV-1 (**b**) and HEK-293T (**c**) cells upon experimental overexpression of 16 anticancer genes. **d**, **e** Cell death in cervical cancer HeLa (**d**) and breast cancer MCF-7 cells (**e**), upon experimental overexpression of 16 anticancer genes. Cells were stained with Dioc_6_ (early apoptosis) and PI (late apoptosis) and subjected to flow cytometry for PI positive and/or Dioc_6_ negative cells. In all cases, luciferase (*luc*) was used for negative control and *CAS2*, *CAS8* and *RIP* were used as positive controls. Histograms represent the average ± standard deviation (SD) of 3 independent transfections. Statistical significance was calculated using two-tailed Student’s t-test (*p ≤ 0.05, **p ≤ 0.005). *pt*: processed transcript, *pr*: partial overlap, *nov*: novel transcript

The plasmids for anticancer genes were obtained from the Japanese National Institute of Technology and Evaluation (NITE) human cDNA library [12]. Cloning of positive and negative controls in pME18SFL3, including *RIPK1*, *CAS2*, *CAS8*, and *GFP* has been described [13, 14]. *tBID* cloned in pcDNA3.1 was a kind gift from Mund’s laboratory [15]. Non-Target pLKO.1 (scrambled) shRNA and pLKO.1 harbouring shRNA against *MYC* were purchased from Sigma-Aldrich, Merck KGaA, Darmstadt, Germany. For low throughput methods, plasmid DNA was purified using Invitrogen’s PureLink plasmid purification kits according to manufacturer’s protocols (Invitrogen, Thermo Fisher Scientific Inc., Waltham, MA USA). For round-1 to round-3 of genetic screening (Fig. 2a), ultra-pure silica oxide large scale plasmid DNA isolation was used [16]. For clonogenic assays, *FBLN5* was excised from the pME18SFL3 vector after *Eco*RI and *Xba*I (FastDigest—Thermo Scientific, Thermo Fisher Scientific Inc.) digestion and cloned into pcDNA 3.1 using standard molecular biology procedures.

### Cell culture

CV-1 [6], HeLa [6], HEK293T [6], MCF-7 [17] and COS-7 cells (a gift from T. Malik, Imperial College London) were cultured in the Dulbecco's Modified Eagle's Medium (DMEM) (with 4500 mg/L glucose, L-glutamine, sodium pyruvate, and sodium bicarbonate, Sigma-Aldrich) supplemented with 10% (v/v) heat inactivated foetal bovine serum (FBS) (Gibco, Thermo Fisher Scientific Inc.).

### Cell transfections

For high-throughput transfections, CV-1 cells were transfected using Xfect transfection reagent (Clontech, Takara Bio Inc. Shiga, Japan) whilst HEK293T cells were transfected using jetPEI transfection reagent (Polyplus-transfection, Strasbourg, France) following instructions provided by the manufacturers. For the low-throughput applications, HeLa cells were transfected using Effectene transfection reagent (Qiagen, Hilden, Germany) whilst MCF-7, CV-1 and COS-7 cells were transfected using Xfect transfection reagent by following the manufacturer’s instructions.

Stably transfected *MYC*-overexpressing CV1 cells were generated by transfecting *cMYC* in pcDNA3.0 using Xfect transfection reagent as above and cells selected initially with 2.5 mg/ml G418 (Sigma-Aldrich) and maintained thereafter with 1.5 mg/mL G418.

Stable downregulation of MYC was obtained by shRNA (in pLKO.1; Sigma-Aldrich) in MCF-7 cells using the Xfect transfection reagent as above. Transfected cells were initially selected with DMEM + 10% FBS containing 1.0 μg/mL puromycin dihydrochloride (Sigma-Aldrich) and thereafter maintained with 0.4 μg/mL puromycin.

### Cell death measurements

#### CPRG assay

For HEK293T cells, it was performed essentially as described [13, 18]. For CV-1 cells, chlorophenol red-β-D-galactopyranoside (CPRG) was added 48 h after transfection and OD at 590 nm was measured after 8 h (CPRG1). The lysis buffer was added 49 h after transfection and OD at 590 nm was measured the following day (CPRG2). CPRG ratio was calculated by dividing 1st OD reading by 2nd OD, i.e., CPRG ratio = CPRG1 / CPRG2.

#### DiOC_6_/propidium iodide staining

Cell death in HeLa and MCF-7 cells was quantified using DiOC_6_/propidium iodide (PI) double staining and flow cytometry [6]. Briefly, 48 h post-transfection, floating and adherent cells were harvested, centrifuged and re-suspended in 150 μL PBS (Dulbecco’s Phosphate Buffered Saline, no calcium, no magnesium, Sigma-Aldrich) containing 40 nM 3,3-dihexaoxacarbocyanine iodide (DiOC_6_) (Life Technologies, Thermo Fisher Scientific Inc.) and 6 μg/mL propidium iodide (PI) (Sigma-Aldrich), incubated at 37 °C for 30 min and further incubated for 30 min at room temperature. Populations of PI-positive and/or DiOC_6_-negative cells were normalised for transfection efficiency using in parallel GFP transfections.

#### Propidium iodide staining

Cell death in CV-1 and CV-1 MYC cells was measured by PI staining. Floating and adherent cells were stained with 20 μg/mL propidium iodide (Sigma-Aldrich) and cell death was calculated as above.

#### Clonogenic cell death assay

For CV-1 cells, 70,000 cells/well were seeded in 6-well plates and transfected the following day. Forty-eight hours post-transfection, media was replaced with fresh one containing 2.5 mg/mL G418 and changed every third day till there were no cells left in the untransfected population (~ 2.5 weeks). Media was removed and the adherent cells were washed with PBS, fixed with 4% (w/v) paraformaldehyde (Sigma-Aldrich), stained with 0.2% (w/v) crystal violet (Sigma-Aldrich) and imaged using a conventional table-top scanner. For COS-7 cells, 20,000 cells/well were seeded in 24-well plates and transfected a day after. Twenty-four hours post-transfection, cells were trypsinized and resuspended in 1 mL DMEM + 10% FBS (final volume). Out of 1 mL cell suspension, 10 µL were re-seeded in the corresponding wells of 6-well plates. Twenty-four hours after re-seeding, media was replaced with fresh one containing 1.0 mg/mL G418 and changed every third day till there were no cells left in the untransfected (‘Untreated’) population (~ 10 days). Cells were fixed and stained with crystal violet as described above.

#### Phenotype inspection for cell death

CV-1 and COS-7 cell were co-transfected with GFP and either FBLN5, luciferase or tBID in 1:4 ratio (1 GFP: 4 test plasmid) in 96-well plates. At 48 h post-transfection, fluorescent green cells were imaged using a IN Cell Analyzer 2000 (GE Healthcare, Chicago,IL, USA) with FITC wavelength filter and counted manually (~ 3000 COS-7 cells and ~ 2500 CV-1 cells).

### Transcriptomic analyses

Cells were seeded in 24-well plates and transfected (for FBLN5 overexpression) as described above. Lysates from 12 wells (100 μL Buffer RLT Plus, Qiagen, per well) were pooled and RNA purified using RNeasy Plus Mini Kit (Qiagen) following manufacturer’s instruction. In addition, optional DNA digestion was performed using On-Column RNase-Free DNase set (Qiagen). Integrity of RNA was confirmed using the RNA Pico Chip (Agilent Technologies, Santa Clara, CA, USA). For each sample, 10 ng of RNA was converted into labelled cDNA using the NuGEN Ovation Pico WTA System V2 (NuGEN, Tecan Group Ltd., Männedorf Switzerland), followed by biotinylating step using the NuGEN Encore® biotin Module (NuGEN). Labelled cDNA was hybridised to Affymetrix GeneChip Clariom D human microarrays (Applied biosystems, Thermo Fisher Scientific Inc.) for 20 h at 45° C, washed, stained (GeneChip Fluidics Station 450) and scanned (GeneChip Scanner 3000 7G) according to the manufacturer’s instructions (NuGEN and Affymetrix). Data processing was conducted using Transcriptome Analysis Console v 4.0.1 (TAC) (Applied biosystems, Thermo Fisher Scientific Inc.). Datasets are available through ArrayExpress [19] accession E-MTAB-11449. Unless otherwise stated, gene expression signatures were filtered through a criteria of fold change (± 2.0) and *p* value and false discovery rate of 0.05 (Fig. 4a). Pathway and comparison analyses were conducted using Ingenuity Pathway Analysis (IPA) [20, 21].

### Cell cycle analysis

Cell cycle was assessed by PI staining as described [6]. Briefly, 20,000 cells/well were seeded in a 24-well plate and cultured for 72 h. Cells were harvested, centrifuged, and re-suspended in 300 μL lysis buffer (20 μg/mL propidium iodide, 0.1% sodium citrate and 0.1% triton X-100 in PBS), incubated for 10 min at room temperature and subjected to flow cytometry. Data analysis was performed using Flowjo v7.6.2.

### MTT assay

Cells were seeded in a 96-well plate and cell proliferation was measured by adding 20 μL of 5 mg/mL MTT solution (Sigma-Aldrich). Following a 3.5 h incubation, MTT solution was replaced with 150 μl of MTT solvent (4 mM HCl, 0.1% NP-40) in isopropanol. The plate was incubated while shaking for 15 min at room temperature, followed by the measurement of OD at 590 nm.

### Live cell imaging

Poly-D-Lysine coated, 35 mm glass bottom dishes (ibidi GmbH, Gräfelfing, Germany) were used for live imaging. Cells were co-transfected with GFP and either FBLN5, luciferase or tBID in 1:4 ratio (1 GFP: 4 test plasmid) as described above and 8 h post-transfection images were captured at 15–20 min intervals. Excitation at 488 nm with 10 mW laser power and 50 ms exposure time was used, emission was detected at 495–550 nm for GFP and halogen lamp for illumination and a full visible spectrum filter cube for phase contrast. During imaging, cells were maintained at 37° C and 5% CO_2_ under high relative humidity in a stage top incubator (Tokai Hit, Shizuoka, Japan) on the Zeiss Elyra wide-field microscope. Image analysis of the Z-stack time series was performed using Fiji [22].

## Results

### Isolation of 16 novel anticancer genes through RISCI

A subset of 377 genes from a pool of genes previously isolated through RISCI from over 30,000 transcripts, which cause cell death in HEK293T cell [13, 14], was used for the genetic screening by transfecting their plasmid DNA along with a β-galactosidase (β-gal) reporter plasmid into CV-1 cells (Fig. 2a). Following the RISCI protocols (one gene per well of a 96-well plate), DNA isolation was performed through the high throughput silica-oxide method [16] whereas cell death was estimated using β-gal /CPRG assay [18]. A total of four 96-well plates were screened for round-1, where 240 out of 377 genes were selected with CPRG ratios less than, or equal to, the upper range of internal negative controls in each 96-well plate (Fig S1). The process was repeated twice with 86 out of 240 genes selected from round-2 and 78 out of 82 genes selected from round-3 (Fig S2). For the subsequent screening, DNA isolation was switched to low throughput miniprep method in order to remove chances of silica-oxide traces interfering with the transfection. After transfection of CV-1 cells in triplicate, 43 out of 78 genes were selected in round-4 of the genetic screen for anticancer genes (Fig S3). These 43 gene candidates were transfected into HEK293T cells and 16 out of 43 genes exhibited significantly higher CPRG ratios than the negative controls (Fig. 2c). This set of 16 genes was tested again in CV-1 cells (Fig. 2b) several times in various settings and designated as anticancer genes for their ability to cause cell death in HEK-293T but not in CV-1 cells (Table 1). These 16 genes were further transfected into HeLa cervical cancer cells and cell death was estimated using the Dioc_6_ + PI combined staining (Fig. 2d); the experimental overexpression of all 16 genes caused significant cell death. The process was repeated in MCF-7 breast cancer cells, where all but one anticancer gene, *LRRFIP2*-partial overlap, triggered cell death (Fig. 2e).

**Table 1:** Novel anticancer genes identified through functional genetic screen

ACG index	Clone ID	GenBank accession number	Gene symbol	Gene ID	Protein coding?
1	1B01	AK074989	*ZNF436-AS1*	ZNF436 antisense RNA	No
2	4G04	AK026893	nd^a^	Novel-ACG4G4 (A novel transcript overlapping to intronic region of *HHLA2*)	nd
3	3C09	AK091900	*SMLR1*	Small leucine-rich protein 1	Yes
4	4G03	AK024890	nd	Novel-ACG4G3 (A novel transcript overlapping to *PYHIN1*)	nd
5	3B03	AK127225	nd	Novel-ACG3B3	nd
6	1A03	AK074632.1	*TMEFF2*	Transmembrane protein with EGF-like and two follistatin-like domains 2	Yes
7	2B08	AK055260	*LINC01529*	Long intergenic non-protein coding RNA 1529	No
8	3B06	AK127945	*HYAL2*	Hyaluronoglucosaminidase 2	Yes
9	2A05	AK056206	*NEIL2*	Nei-like DNA glycosylase 2	Yes
10	1B08	AK075147	*FBLN5*	Fibulin 5	Yes
11	2H03	AK124577	*YPEL4*	Yippee-like 4	Yes
12	4E06	AK024927.1	nd	Novel-ACG4E6 (ENSG00000261116 lncRNA- overlapping to *FAM83B*)	No
13	3D05	AK092480	*PHKA2*	Non-coding, processed transcript	No
14	4F06	AK024692	*LRRFIP2*	Leucine rich repeat (in FLII) interacting protein 2. Partial overlap—last seven exons	nd
15	4F04	AK024913	nd	Novel-ACG4F4 (A novel transcript overlapping to *CCDC6*)	nd
16	4D09	AK024690	nd	Novel-ACG4D9 (A novel transcript overlapping to *FBXL20*)	nd

^a^*nd* not determined

### Identification of novel anticancer genes

The NITE cDNA library used for screening [12, 14] contains both protein coding and non-coding sequences, cloned predominantly in pME18SFL3 shuttle vector. A unique GenBank [23] accession number (AK number) identifies each cDNA clone and also provides its DNA sequence. In order to establish the identities of 16 novel anticancer genes, we sequenced their plasmid DNA and matched the sequences to those provided by NITE [12]. The latter was subjected to BLAST-like Alignment Tool (BLAT) [24] using the Ensembl genomic interpretation system [25]. Clone 1B01 cDNA sequence overlapped with the exons and introns of *ZNF436-AS1*, whereas clones 3C09, 1A03, 2B08, 3B06, 2A05, 1B08, 2H03 and 3D05 overlapped with the exons of *SMLR1*, *TMEFF2, LINC01529, HYAL2, NEIL2, FBLN5, YPEL4* and *PHKA2-*processed transcript respectively. For clone 4F06, the query cDNA sequence overlapped with only the last 7 exons of *LRRFIP* splice variant 002. Clone 3B03 appeared to include intergenic sequences which overlapped specific chromosomal regions, thus exhibiting a peculiar structure typical of exons (Fig S4a). Clone 4E06 overlapped a novel non-coding RNA transcript, ENST00000562834, for which the gene identity remains undefined (Fig S4b). Clones 4G04, 4G03, 4F04 and 4D09 only overlapped with small regions of the exons or the introns of some previously identified genes (Fig S4c-f). The genes with undetermined identities were hereafter designated as ACGs (anticancer genes) (Table 1).

### *FBLN5* as a novel anticancer gene

**Fig. 3:**
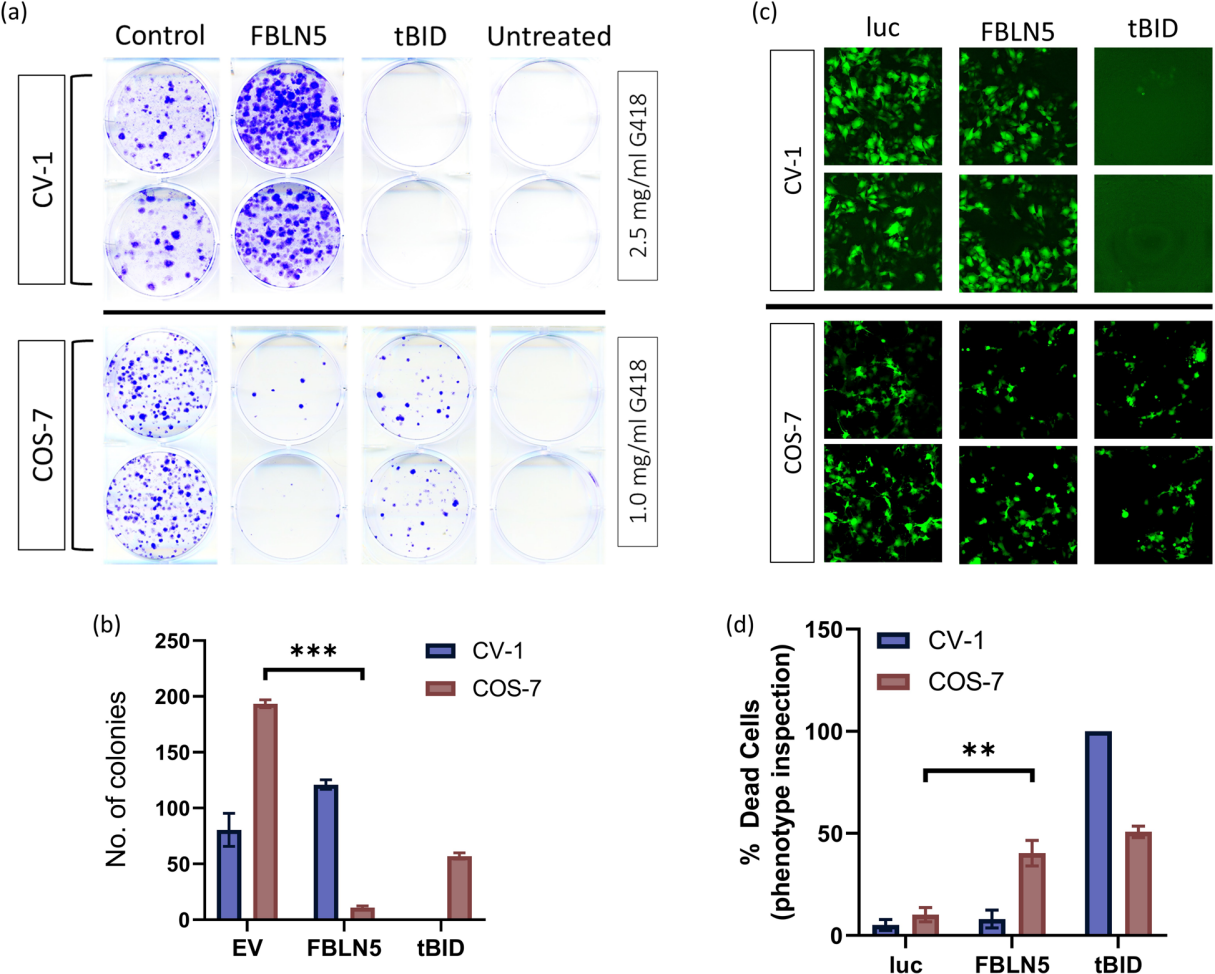
FBLN5 induces cell death in transformed COS-7 cells but not in normal CV-1 cells. **a**, **b** Clonogenicity assay upon stable overexpression of *FBLN5* in CV-1 and COS-7 cells. *FBLN5*, *tBID* (positive control) and pcDNA 3.1 empty vector (negative *control)* were transfected into CV-1 and COS-7 cells, selected and maintained in 2.5 mg/mL and 1.0 mg/mL G418 containing media, respectively. When there were no cells left in the untransfected (*untreated*) population, plates were fixed with 4% PFA, and colonies stained with crystal violet and counted. Histogram (**b**) represents average ± SD number of colonies in the representative image (**a**) with two independent transfections. **c**, **d** cell death in CV-1 and COS-7 cells upon *FBLN5* transfection as determined by phenotypic inspection. CV-1 and COS-7 cell were co-transfected with GFP and either FBLN5, luciferase (*luc,* negative control) or tBID (positive control) in 1:4 ratio (1 GFP: 4 test plasmid) and 48 h post-transfection, fluorescent green cells were imaged (**c**) and manually counted (~ 3000 COS-7 cells and ~ 2500 CV-1 cells; (**d**)). Rounded, clustered and/or cells with disfigured morphology were considered dead. No green cells could be detected in CV-1 cells transfected with *tBID* and 100% was added manually in the chart. **c** indicates a representative image from more than 75 fields of view. Histogram (**d**) represents average ± SD from three independent transfections. Statistical significance was calculated using two-tailed Student’s t-test (**p ≤ 0.005, ***p ≤ 0.0005)

Among the novel anticancer genes, *FBLN5*, encoding a secreted glycoprotein belonging to a small but versatile family of secreted extracellular matrix proteins known as fibulins [26], has a role in numerous cancers [27]. Being a secreted protein, FBLN5 offers prospects for tumour-specific protein therapy. FBLN5 exhibited consistent induction of cell death through all stages of screening and was thus chosen for further proof-of-principle investigation. In order to avoid potential challenges posed by the apparent genetic differences between HEK293T and CV-1 cells in deciphering the differences in function of FBLN5 between the two systems, COS-7 cells were used as a model for the transformed cell line as these cells were originally generated by transforming CV-1 cells with SV40-origin defective DNA [11]. COS-7 and CV1 cells were transfected with *FBLN5* and cell death determined by clonogenicity assay (Fig. 3a). In comparison to negative control, the experimental overexpression of FBLN5 decreased the number of COS-7 colonies dramatically whilst CV-1 colonies remained largely unaffected (Fig. 3b). These results were confirmed by phenotypic inspection of CV-1 and COS-7 cells upon co-transfection of *FBLN5* along with a GFP reporter plasmid. Rounded, clustered and/or cells with disfigured morphology were considered dead (Fig. 3c). No obvious cell death was observed in CV-1 cells upon the experimental overexpression of FBLN5; however, FBLN5 caused significant cell death in COS-7 cells (Fig. 3d). Interestingly, the positive control tBID eradicated CV-1 cells upon overexpression, whereas COS-7 cells remained relatively resistant to this potent apoptosis inducer. Additionally, the morphological changes associated with FBLN5-induced cell death in COS-7 cells were confirmed through high-resolution time-lapse microscopy (Supplementary data M1, M2, M3).

### Transcriptomic analysis upon *FBLN5* ectopic expression

**Fig. 4:**
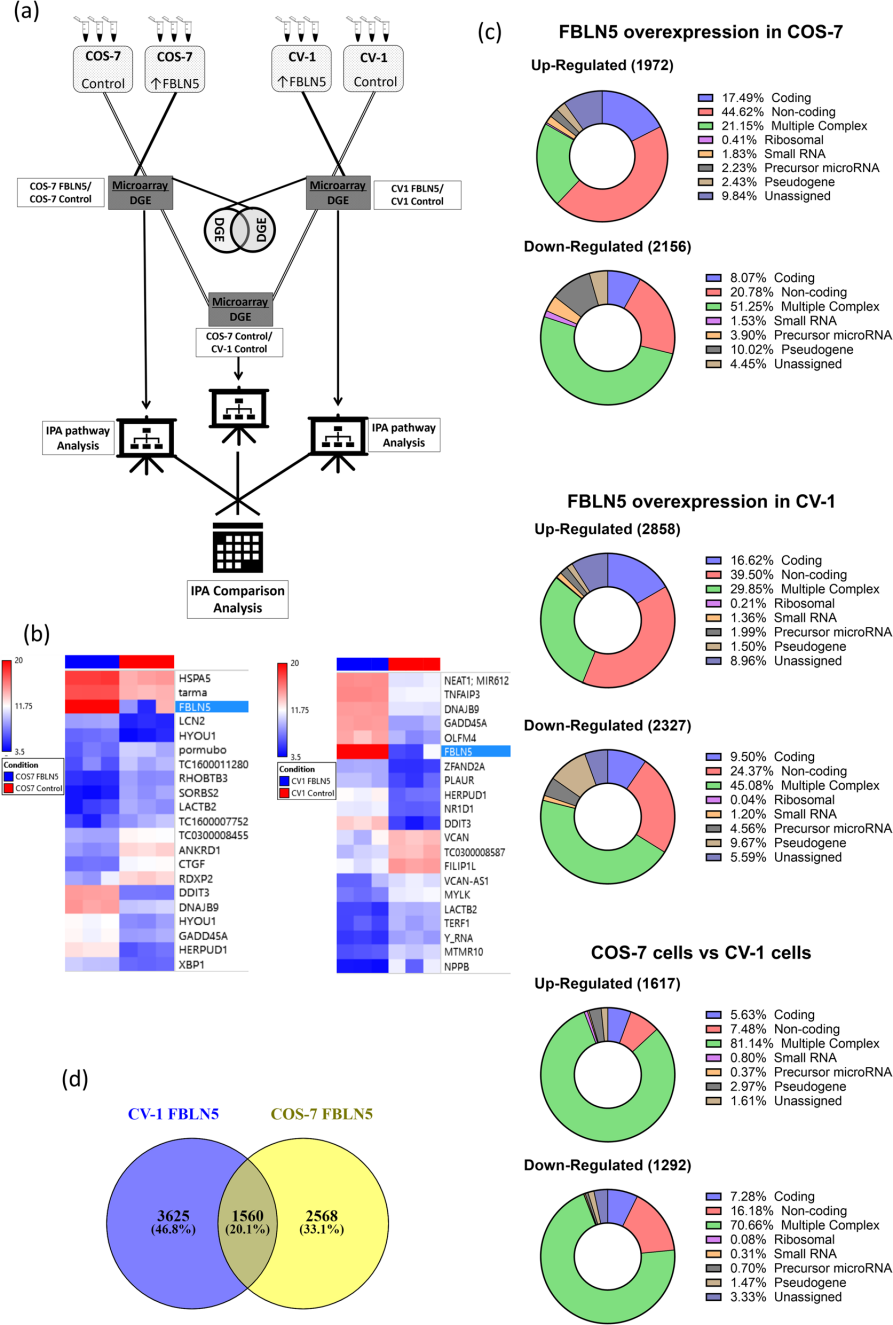
Differential transcriptomic analysis of CV-1 and COS-7 upon FBLN5 experimental overexpression. **a** Schematic representation of the comparative transcriptomic analysis in CV-1 and COS-7 cells upon FBLN5 overexpression. Wild type and FBLN5 overexpressing CV-1 and COS-7 cells were subjected to microarray hybridization and subsequent differential gene expression analysis (DGE). Three sets of differentially expressed genes were obtained: CV-1 transfected with FBLN5 / wild type CV-1, COS-7 transfected with FBLN5 / wild type COS-7 and wild type COS-7 / wild type CV-1. These three sets were subjected to the Ingenuity Pathway Analysis (IPA) Core Analysis individually. The three core analysis reports thus obtained were subjected to the IPA Comparison Analysis. As an additional step, the two differentially expressed sets of genes (CV-1 transfected with FBLN5 / wild type CV-1 and COS-7 transfected with FBLN5/ wild type COS-7) were also compared separately using Venn diagram. **b** Heatmap of 20 most differentially expressed genes between *FBLN5*-transfected COS-7 (left) and CV-1 (right) cells *vs* wild type COS-7 and CV-1 cells respectively. Heatmap filters, COS-7 cells: -8.5 ≤ fold change ≥ 7, *p* ≤ 0.05, FDR ≤ 0.05, CV-1 cells: -10.8 ≤ fold change ≥ 13.5, *p* ≤ 0.05, FDR ≤ 0.05. **c** Types of differentially expressed transcripts in COS-7 cells transfected with *FBLN5* / wild type COS-7 cells (top), CV-1 cells transfected with *FBLN5* / wild type CV-1 cells (mid) and wild type COS-7 cells / wild type CV-1 cells (bottom). Filter: -2 ≤ fold change ≥ 2, *p* ≤ 0.05, FDR ≤ 0.05. **d** Comparative Venn diagram of differentially expressed genes in CV-1 cell (blue) and COS-7 (yellow) upon *FBLN5* transfection, only 20.1% differentially expressed genes were common among the two differentially expressed gene sets

In order to gain a genome-wide view of the differential functional effects of FBLN5, we performed transcriptomic analysis of *FBLN5*-transfected CV-1 and COS-7 cells (Fig. 4a). The analysis generated three separate groups of differentially expressed gene sets (DEGS), i.e., the transcriptomic changes between control COS-7 cells and those transfected with *FBLN5* (FBLN5 COS-7 / wild type COS-7), wild type CV-1 cells and the CV-1 cells transfected with *FBLN5* (FBLN5 CV-1 / wild type CV-1) and between wild type COS-7 cells and wild type CV-1 cells (wild type COS-7 / wild type CV-1). Interestingly, despite having approximately half the expression level (Fig S5), FBLN5 overexpression in CV-1 cells resulted in a higher number of differentially expressed genes (5,185 transcripts) than in FBLN5 overexpressing COS-7 cells (4,128 transcripts) (Fig. 4c). The set of differentially expressed genes between wild type COS-7 and wild type CV-1 cells was smaller (2,909 transcripts) than the differentially expressed genes upon FBLN5 overexpression in either CV-1 or COS-7 cells. Also, FBLN5 overexpression resulted in changes in the expression of different sets of genes in CV-1 and COS-7 cells; only 20.1% of transcripts were common between FBLN5 overexpressing CV-1 and COS-7 cells (Fig. 4d, Fig S6).

### Gene ontology comparison analysis

**Fig. 5:**
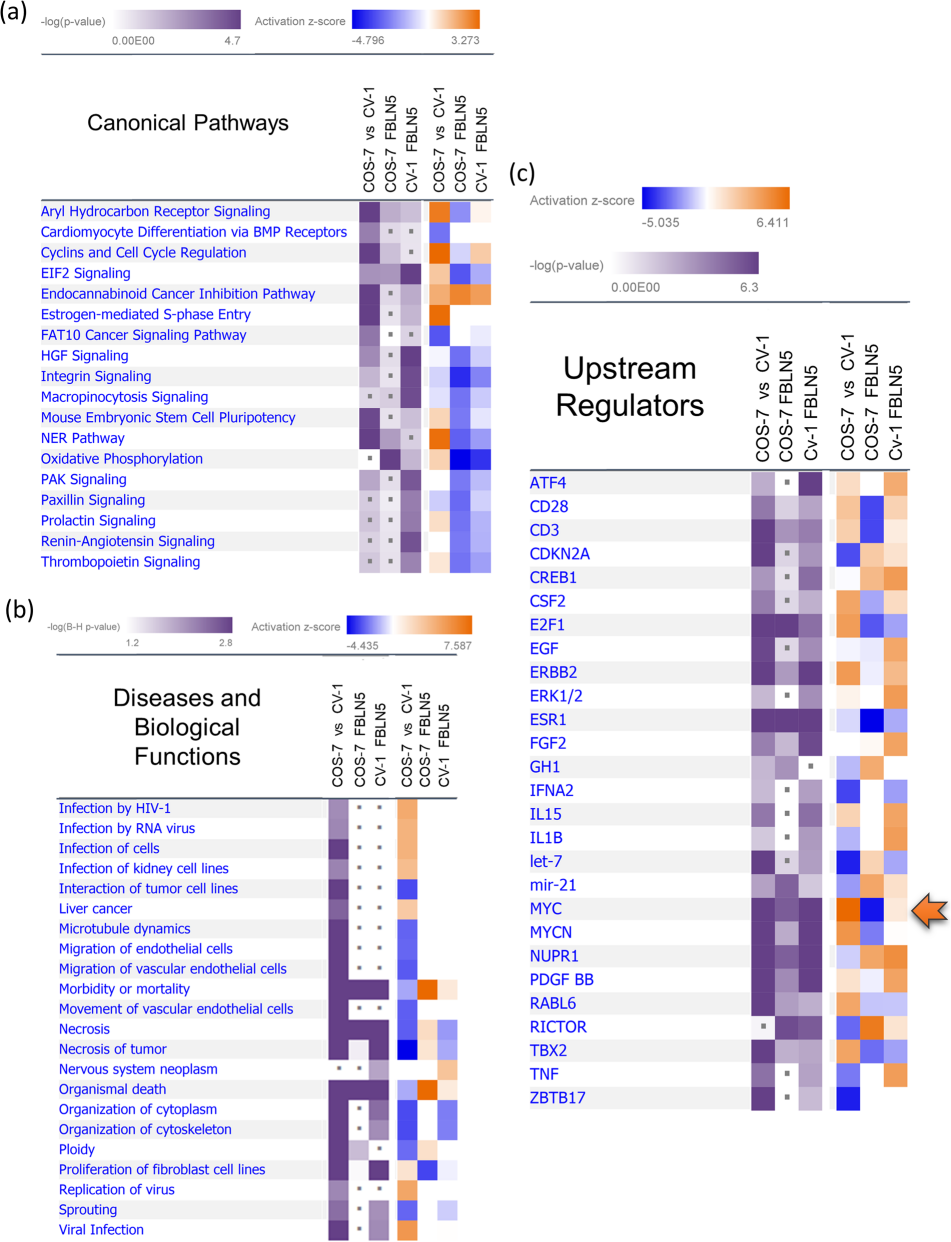
FBLN5 overexpression leads to inhibition of MYC in COS-7 cells but not in CV-1 cells. **a**-**c** IPA Comparative pathways analysis between the differentially expressed gene sets in wild type COS-7 cells *vs* wild type CV-1 cells (*COS-7 vs CV-1*), *FBLN5*-transfected COS-7 *vs* wild type COS-7 cells (*COS7 FBLN5*) and *FBLN5*-transfected CV-1 *vs* wild type CV-1 cells (*CV1 FBLN5*). The dot represents -log_10_(*p*-value) ≤ 1.5 (below significance threshold). **a** IPA Canonical Pathways comparison analyses, heatmap cut off values: -log_10_(*p*-value) ≥ 1.3, -log_10_(B-H (Benjamini-Hochberg) *p*-value) ≥ 2 and activation z-score ≥ 2.5. **b** IPA Downstream Effects comparison analyses, heatmap cut off: -log_10_(*p*-value) ≥ 1.3, -log_10_(B-H *p*-value) ≥ 1.3 and activation z-score ≥ 2.5. **c** IPA Upstream Regulator comparison analyses, heatmap cut off: -log_10_(*p*-value) ≥ 1.3, -log_10_(B-H *p*-value) ≥ 1.7 and activation z-score ≥ 3.5. The predicted upstream regulation of MYC between three conditions is pointed out. A differential gene expression analysis filter comprising fold change ≥ 2 and ≤ -2, *p* ≤ 0. 05 and FDR ≤ 0.05 was used in all cases

Each DEGS (‘wild type COS-7 cells *vs* wild type CV-1 cells’, ‘COS-7 cells transfected with FBLN5 *vs* wild type COS-7 cells’ and ‘CV-1 cells transfected with FBLN5 *vs* wild type CV-1 cells’) was analyzed using the IPA Core Analysis [20, 21] and then subjected to the IPA Comparison Analysis (Fig. 4a) where the results of the Canonical Pathways analysis, the Upstream Regulators Analysis and the Downstream Effects Analysis were compared among the three datasets (Fig. 5). The Upstream Regulator Analysis predicts the causative factors for the given transcriptomic changes and the Downstream Effects analysis predicts the same for downstream changes based on the DEGS provided [20]. The DEGS in all three datasets overlapped with a diverse range of canonical pathways which appeared mostly unrelated to each other (Fig. 5a). Moreover, SV40 transformation (wild type COS-7 *vs* wild type CV-1 cells) and FBLN5 overexpression in CV-1 cells dominated the canonical pathways overlaps (Fig. 5a). Upon FBLN5 overexpression, only three pathways, *aryl hydrocarbon receptor signalling*, *EIF2 signalling* and *oxidative phosphorylation*, showed statistically significant overlap in both COS-7 and CV-1 cells. The results of the Downstream Effects comparison analysis (Fig. 5b) indicated the activation of diseases and functions pertaining to neoplastic transformation and viral infection in wild type COS-7 *vs* wild type CV-1 cells, which include *liver cancer*, *replication of virus*, *infection by RNA virus* and *viral infection*. Similarly, *morbidity or mortality*, *necrosis*, *necrosis of tumour* and *organismal death* were predicted to be inhibited upon SV40 transformation whereas the same processes were predicted to be activated when FBLN5 was experimentally overexpressed in COS-7 cells. On the other hand, these processes were either being inhibited or negligibly activated in *FBLN5*-transfected CV-1 cells. For example, among the molecules involved in *morbidity or mortality*, *CASP7* (caspase 7) was upregulated by 2.07-fold upon FBLN5 overexpression in COS-7 cells, whereas its mRNA levels remained unaffected either upon SV40 transformation or FBLN5 overexpression in CV-1 cells (data not shown — transcriptomic datasets are available through ArrayExpress accession E-MTAB-11449).

The Upstream Regulator Comparison Analysis (Fig. 5c) predicted the pro-proliferation regulators like *CD28*, *CD3* and *MYC* to be activated upon SV40 driven neoplastic transformation. These regulators were inhibited in FBLN5 overexpressing COS-7 cells and on the other hand, the activity of these growth promoting regulators was either unaffected or slightly enhanced in FBLN5 overexpressing CV-1 cells. Among the regulators exhibiting this trend, *MYC* had the highest -log_10_(*p*-values) and the difference in activation z-scores among the *FBLN5* transfected CV-1 and COS-7 cells, where *MYC* was predicted to be most potently inhibited by *FBLN5* in COS-7 cells (Fig S7) with a slight activation in CV-1 cells (Fig S8). Through filtering the results of upstream regulator analysis (-log_10_(*p*-value) ≥ 1.3, activation z score ≥ 3.5 and -log_10_(B-H adjusted *p*-value) ≥ 1.7), we extracted a subset of the most significant upstream regulators in all the three tested conditions, i.e., COS-7 *vs* CV-1, COS-7-FBLN5 and CV1-FBLN5. However, *TP53* and *E2F1* were among the topmost upstream regulators in the DEGS between wild type COS-7 cells and wild type CV-1 cells (Supplementary Table -TS1).

### *FBLN5* as a synthetic lethal partner of *MYC*

**Fig. 6:**
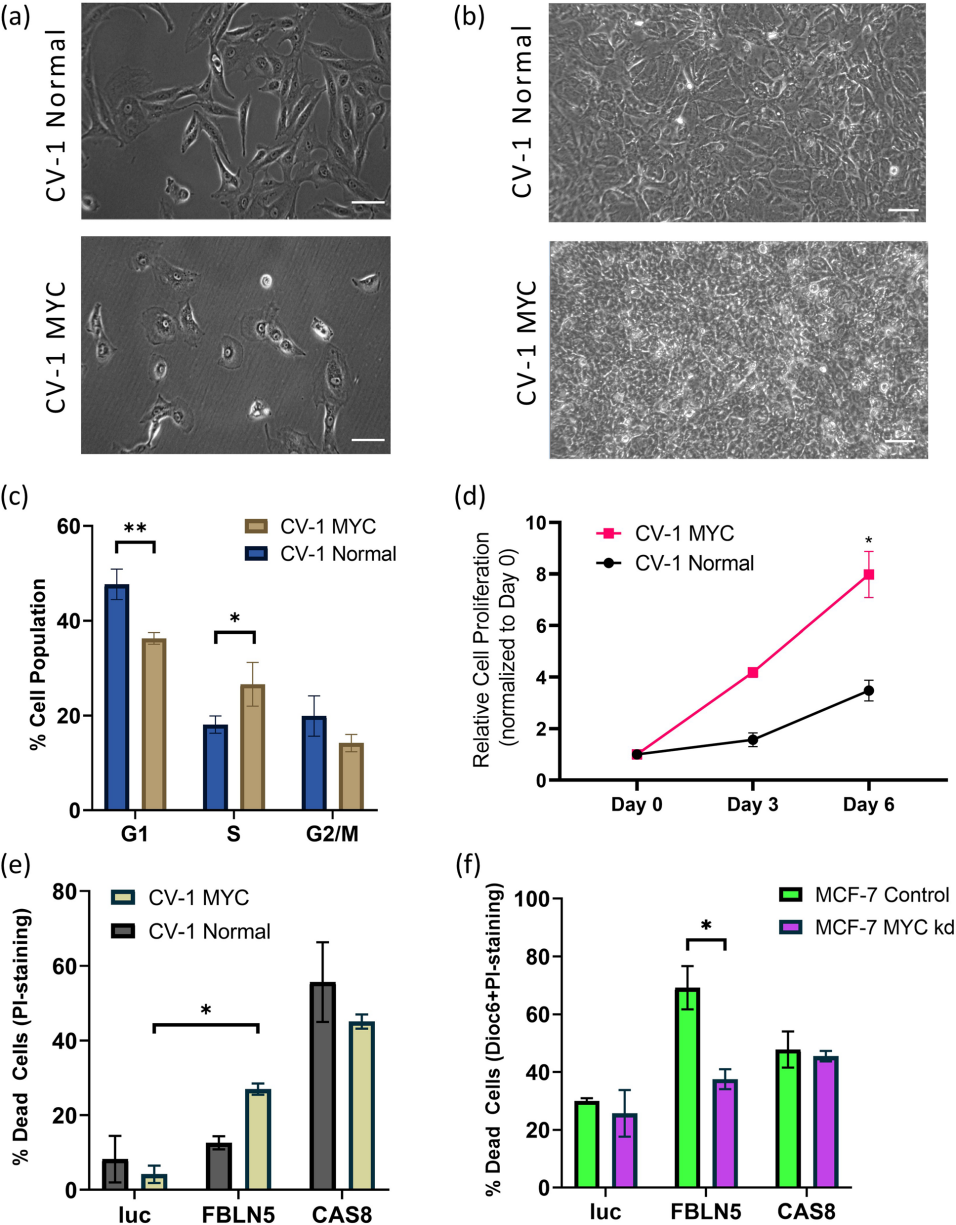
*FBLN5* as synthetic lethality partner of *MYC*. **a**-**d** Neoplastic transformation of CV-1 cells upon stable overexpression of MYC. **a** Phase contrast micrographs of normal CV-1 cells and MYC-overexpressing CV-1 cells (*CV-1 MYC*). **b** Loss of contact inhibition in CV-1 MYC cells. Phase contrast micrographs of 3 weeks-old cell cultures. (a-b) Scalebar = 80 μm. **c** Cell cycle distribution of CV-1 normal cells and CV-1 MYC cells after PI staining and analysed by flow cytometry. **d** Growth curves of CV-1 normal cells and CV-1 MYC cells in serum-free medium. Cell proliferation was determined by MTT colorimetric assay with absorbance at 590 nm normalized to CV-1 normal cells at day 0. **e** Cell death in both normal CV-1 and CV-1 MYC cells upon *FBLN5* transfection. Cells were harvested 48 h post-transfection, stained with PI and subjected to flow cytometry. Luciferase (*luc*) was used as a negative control and *CAS8* was used as a positive control. **f** Cell death in MYC knockdown (MYC shRNA stable) MCF-7 cells upon *FBLN5* transfection. MCF-7 cells stably transfected with scrambled shRNA were used as control (*MCF-7 Control*). Cells were harvested 72 h post-transfection, stained with Dioc_6_ and PI and subjected to flow cytometry. Luciferase (*luc*) was used as a negative control and *CAS8* was used as a positive control. Numerical values represent average ± SD of 3 independent transfections. Statistical significance was calculated using two-tailed Student’s t-test (*p ≤ 0.05, **p ≤ 0.005)

Our transcriptomic data and the pivotal roles of *MYC* in cancer and *FBLN5* regulation [28, 29] led us to hypothesize that *MYC* might be a synthetic lethality partner of *FBLN5*. In order to test this hypothesis, we generated CV-1 cells stably transfected with *MYC* (CV-1 MYC cells) which exhibited traits typical of neoplastic transformation including change in morphology (Fig. 6a), loss of contact inhibition (Fig. 6b), shift in cell cycle distribution to S phase (Fig. 6c) and growth factor independent proliferation (Fig. 6d). In order to test the effects of FBLN5 expression on these cells, we transfected CV-1 MYC cells and native CV-1 cells with *FBLN5* and determined cell death by flow cytometry after PI staining. Experimental overexpression of FBLN5 led to a 24.4% increase in cell death in CV-1 MYC cells relative to negative control (*luc*) whereas in native CV-1 cells, *FBLN5* transfection caused a non-statistically significant increase of 4.3% in cell death pointing towards *MYC* being a synthetic lethality partner of *FBLN5*. To substantiate these results further, we knocked down *MYC* expression using shRNA by 40% in breast cancer MCF-7 cells, a cell line which expresses high levels of *MYC* [30], (Fig S9). Flow cytometric analyses of early (Dioc_6_ staining) and late (PI staining) apoptosis showed a 31.6% reduction in FBLN5-induced cell death upon *MYC* knockdown (Fig. 6f). These data indicate that *FBLN5* acts as a synthetic lethality partner of *MYC*.

## Discussion

We have isolated 16 novel anticancer genes through a gain of function forward genetic screen employing RISCI protocols. Gain of function screening, in which an activating signal is induced by ectopically overexpressing a gene into the cells, offers several advantages over the conventional loss of function screening strategies using siRNA, shRNA or CRISPR-Cas9-based silencing of endogenous genes. Firstly, the activating signal induced by overexpressing an exogenous gene cannot be functionally compensated or replaced by any endogenous gene. Secondly, ectopically overexpressed genes can activate signalling pathways and reveal important functional information, whereas in loss of function screens knocked down genes often impart redundant functions, or their functional contribution is too small to be detectable. Additionally, we used untransformed isogenic cells, while the conventional approach uses cancer cell lines where isogenicity is achieved through stable knockdown of individual genes. Attaining the functional redundancy of a targeted gene using RNA interference is not only challenging (especially for genes with low constitutive expression), but the dynamics of gene integration and RNA metabolism can also cause non-specific functional effects. Nevertheless, we used HeLa and MCF-7 cells after the isolation of anticancer genes in CV-1 cells to confirm their ability to impart cell death in cancer cells.

The protein-coding novel anticancer genes have a wide range of functional spectra: *HYAL2* is a ubiquitous extracellular matrix hyaluronan-degrading enzyme [31], *NEIL2* is involved in DNA base excision repair [32], *YPEL4* is involved in cell proliferation [33], *TMEFF2* is commonly methylated in numerous cancers and it is involved in diverse processes including neuroprotection [34]. *ZNF436-AS1*, *LINC01529* and *PHKA2* processed transcripts are *bona fide* non-coding RNAs. In addition, *ACG4E6* sequence overlaps a non-coding RNA transcript which lies within an exon of protein coding gene *FAM83B* (Fig S4b). Similarly, the sequences for *ACG4F4* and *ACG4D9* lie within single exons of protein coding genes *CCDC6* and *FBXL20*, respectively, thus leading to the possibility of these transcripts being non-coding genes as well (Fig S4e-f). The sequence of *ACG4G4* overlapped to an intronic region of a protein coding gene *HHLA2* (Fig S4c)*,* as microRNA precursors are commonly found within the introns and intergenic locations of protein-coding genes [35], *ACG4G4* could be the precursor sequence for a novel microRNA [36]. Although *ACG3B3* exhibited the intergenic sequences typical of exons (Fig S4a), its sequence did not match with any previously annotated transcript on the comprehensive GENCODE 38 database and could represent a novel gene.

Ideally primary human cells should be used to screen for anticancer genes, however, prolonged doubling times, fastidious growth conditions and fragility of primary cell cultures render them unsuitable for high throughput screening. Relative inability of these cells to take up plasmid DNA poses an additional hurdle as consistency of transfection and functional levels of gene overexpression are necessary for a gain for function screens and validation. Use of xenograft models offer similar challenges in terms of gene overexpression. Capitalizing on the results of our previous studies [6, 10], we adopted CV-1 cells as a model of normal cells as no defect in tumour suppressor genes or overexpression of oncogenes that would drive cellular transformation has been reported. An established transformed version of CV-1 cells (COS-7 cells) offers a transformed biological system with minimal core genetic differences, thus enabling the investigation of differential functioning of an anticancer gene through transcriptomic analysis. The genomes and transcriptomes of human and vervet monkey are known to be exceptionally similar. Warren et al. [37] reported an average coverage of 98.8% upon aligning 97.9% of 46,823 human known RefSeq transcripts to the vervet genome.

In order to gain further insights in the mechanism of action of anticancer genes, we selected *FBLN5* as a proof of concept. Various cell death assays were tested in COS-7 cells including Dioc_6_/PI staining, TMRE/DRAQ7 staining and Annexin V-FITC/7AAD staining (data not shown), but a sufficient signal to background ratio could not be achieved. We hypothesize that the exceptionally high division rate of COS-7 cells makes the untransfected population of cells surpass the sensitivity threshold of these conventional cell-death assays. We therefore adapted an unconventional approach of assaying cell death where *FBLN5* was cloned into pcDNA 3.1 (with neomycin resistance cassette), transfected into the CV-1 and COS-7 cells and selection pressure applied by G418. As expected, FBLN5 caused extensive cell death in COS-7 but not in CV-1 cells (Fig. 3a-b). Additionally, expression of the apoptosis inducer tBID completely eradicated CV-1 cells, as the cell death circuitry remains largely intact in these untransformed cells, although it had little effect on COS-7 cells. The results were corroborated through phenotypic inspection (Fig. 3c-d), also used in other studies using transfected COS-7 cells [38–41], with morphological changes confirmed by high-resolution time-lapse microscopy (Supplementary data M1, M2, M3). Moreover, processes like *morbidity and mortality* and *organismal death* were strongly activated in *FBLN5* transfected COS-7 cells but not in CV-1 cells (Fig. 5b). FBLN5 overexpression in COS-7 and CV-1 cells resulted in modulation of expression of different sets of genes, thus eliminating the possibility of this differential effect being an artefact due to differences in *FBLN5* transfection or overexpression (Fig. 4d, Fig S6).

The Upstream regulator comparison analysis identified *MYC* as an important player in the transformation of CV-1 cells to COS-7 cells (Fig. 5c). Corroborating this bioinformatic analysis, CV-1 cells stably transfected with MYC exhibited classical traits of neoplastic transformation, some of which have already been reported [42, 43] (Fig. 6a-d). Additionally, the transcriptomic and gene ontology analyses indicated FBLN5-induced selective cell death in COS-7 cells involved a potent inhibition of *MYC*. Indeed, cell death was observed in CV-1 MYC cells upon experimental overexpression of FBLN5, thus indicating *MYC* being the synthetic lethality partner of *FBLN5*. This premise is further reinforced by loss of FBLN5-induced cell death in MCF-7 cells upon MYC knockdown. In addition to being a versatile oncogenic factor, *MYC* can also act as a strong apoptosis inducer [44]. On the other hand, the induction of cell death upon knockdown of *MYC* has also been reported [45, 46]. The mechanisms involved in *MYC* inhibition (or downregulation)-derived cell death are not currently known and warrant further investigation.

FBLN5 is positively regulated by MYC [29], on the other hand FBLN5 has been reported to downregulate MYC through inhibition of β-catenin signalling both in vivo and in vitro models of metastatic lung cancer [47]. The synthetic lethal relationship between FBLN5 and MYC need further investigation. Higher order mechanistic network analyses followed by functional studies could be employed to decipher the possible links between FBLN5, MYC and cell death.

## Conclusions

A small-scale genetic screen has identified 16 novel anticancer genes. This suggests the possibility of numerous tumour-specific cell death inducers being present in human genome. Further genome-wide screens employing similar methodologies (the combination of RISCI with gain of function forward screening approach) could identify several novel anticancer genes. FBLN5 was chosen as proof of principle and identified as a synthetic lethal partner of *MYC*. Dedicated synthetic lethal screens can be used to isolate anticancer genes directed against specific oncogenic drivers. Anticancer genes could not only reveal novel oncogenic and onco-sustaining factors but, together with advances in gene therapy and tumour directed delivery systems, novel anticancer genes could prove powerful targeted cancer therapy tools.

### Supplementary Information

**Additional file 1: Supplementary Figure S1.** Round-1 of genetic screen for anticancer genes. **Supplementary Figure S2.** Round-2 and round-3 of genetic screen for anticancer genes. **Supplementary Figure S3.** Round-4 of genetic screen for anticancer genes. **Supplementary Figure S4.** Ensembl BLAT analysis of novel anticancer genes. **Supplementary Figure S5.** Confirmation of FBLN5 overexpression in triplicate samples for transcriptomic analysis. **Supplementary Figure S6.** Variance based principal component analysis depicting all 12 samples used in the transcriptomic analysis of wild type and FBLN5-transfected CV-1 and COS-7 cells. **Supplementary Figure S7.** Network view of MYC as the predicted upstream regulator for the transcriptomic changes observed upon FBLN5 overexpression in COS-7 cells. **Supplementary Figure S8.** Network view of MYC as the predicted upstream regulator for the transcriptomic changesobserved upon FBLN5 overexpression in CV-1 cells. **Supplementary Figure S9.** Confirmation of stable MYC knockdown in MCF-7 cells. **Supplementary Table TS1.** log_10_(Benjamini-Hochberg *p*-value) and z-score for the upstream regulator comparison analysis with 5 highest -log_10_(*p*-values) in COS-7 vs CV-1 dataset.

**Additional file 2: Supplementary Movie M1.** Time lapse series micrographs of COS-7 cells transfected with GFP and FBLN5 in 1:4 ratio.

**Additional file 3: Supplementary Movie M2.** Time lapse series micrographs of COS-7 cells transfected with GFP and luciferase (negative control) in 1:4 ratio.

**Additional file 4: Supplementary Movie M3.** Time lapse series micrographs of COS-7 cells transfected with GFP and tBID (positive control) in 1:4 ratio.

## Data Availability

Transcriptomic datasets are available through ArrayExpress accession E-MTAB-11449. Other datasets related to this paper can be provided by the lead author upon reasonable request.

## Abbreviations

ACG: Anticancer gene
β-gal: β-Galactosidase
B-H: Benjamini-Hochberg
BLAT: BLAST-like alignment tool
CPRG: Chlorophenol red-β-D-galactopyranoside
DEGS: Differentially expressed gene set
DiOC_6_: 3,3-Dihexaoxacarbocyanine iodide
DMEM: Dulbecco’s modified Eagle’s medium
IPA: Ingenuity Pathway Analysis
PBS: Phosphate buffered saline
PI: Propidium iodide
NITE: Japanese National Institute of Technology and Evaluation
RISCI: Robotic single cDNA investigation
TAC: Transcriptome Analysis Console
