# Supplementary material for: Genetic screening for anticancer genes highlights FBLN5 as a synthetic lethal partner of MYC
Source: Cell Commun Signal. 2023 Oct 20;21:295. doi: 10.1186/s12964-023-01300-3 (PMC10588048; doi:10.1186/s12964-023-01300-3)
Supplement: Supplementary file 2 — Additional file 1: Supplementary Figure S1. Round-1 of genetic screen for anticancer genes. Supplementary Figure S2. Round-2 and round-3 of genetic screen for anticancer genes. Supplementary Figure S3. Round-4 of genetic screen for anticancer genes. Supplementary Figure S4. Ensembl BLAT analysis of novel anticancer genes. Supplementary Figure S5. Confirmation of FBLN5 overexpression in triplicate samples for transcriptomic analysis. Supplementary Figure S6. Variance based principal component analysis depicting all 12 samples used in the transcriptomic analysis of wild type and FBLN5-transfected CV-1 and COS-7 cells. Supplementary Figure S7. Network view of MYC as the predicted upstream regulator for the transcriptomic changes observed upon FBLN5 overexpression in COS-7 cells. Supplementary Figure S8. Network view of MYC as the predicted upstream regulator for the transcriptomic changesobserved upon FBLN5 overexpression in CV-1 cells. Supplementary Figure S9. Confirmation of stable MYC knockdown in MCF-7 cells. Supplementary Table TS1. log10(Benjamini-Hochberg p-value) and z-score for the upstream regulator comparison analysis with 5 highest -log10(p-values) in COS-7 vs CV-1 dataset. [file 12964_2023_1300_MOESM1_ESM.pdf]

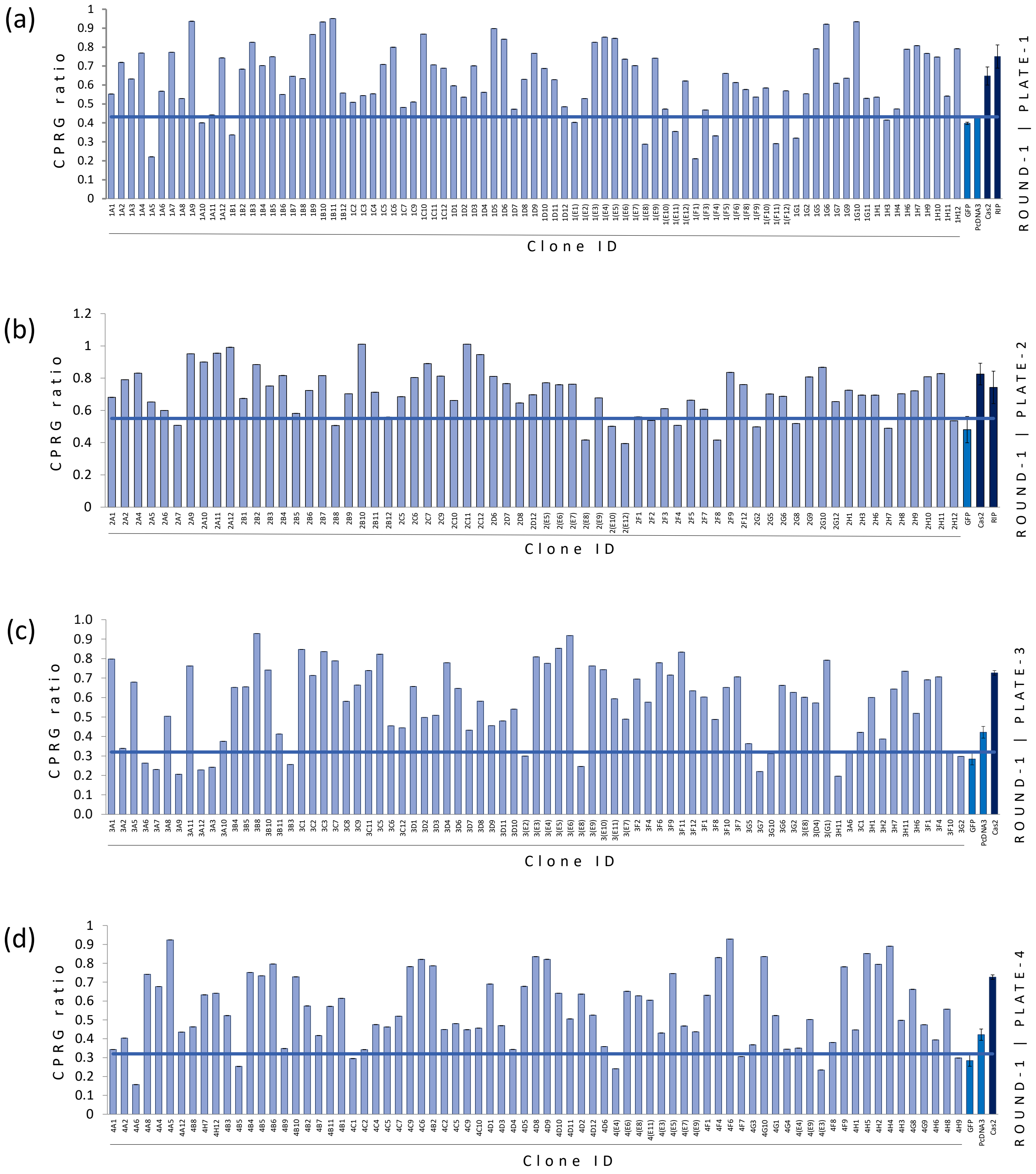

Supplementary Figure S1: Round-1 of genetic screen for anticancer genes. A subset of 377 cell-death inducers were co-transfected with a β-Gal-expressing plasmid in CV-1 cells in four 96-well plates (a), (b), (c) and (d) – one gene per well. Plasmid DNA was purified using a silica-oxide method and relative cell death was determined by CPRG assay. *GFP* and pcDNA3 were used as negative controls while *CAS2* and *RIP* were used as positive controls. The horizonal line drawn at the upper range of negative control was used as selection threshold. Genes with CPRG ratios equal or below threshold were selected for the subsequent rounds of screening.

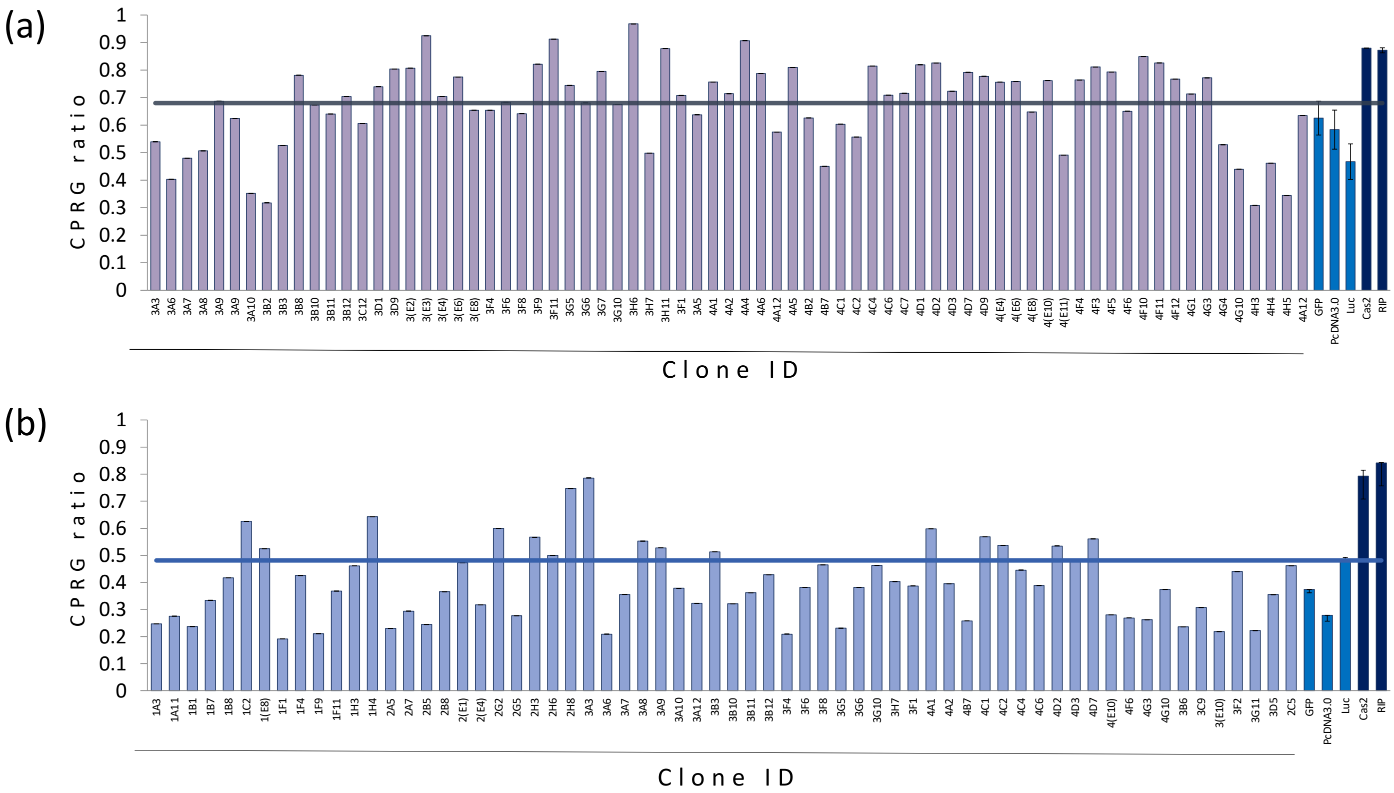

Supplementary Figure S2: Round-2 and round-3 of genetic screen for anticancer genes. (a) Round-2: representative results from 240 gene candidates obtained in round-1. Eighty-six genes with CPRG ratios equal or below the upper range of the negative control (horizontal line) were selected for subsequent screening. (b) Round-3: out of 86 candidate genes selected from round-2, 78 genes with CPRG ratios equal or below the upper range of the negative control (horizontal line) were selected for the subsequent screening. In all cases, *GFP*, luciferase (*Luc*) and pcDNA3 were used as negative controls whilst *CAS2* and *RIP* were used as positive controls.

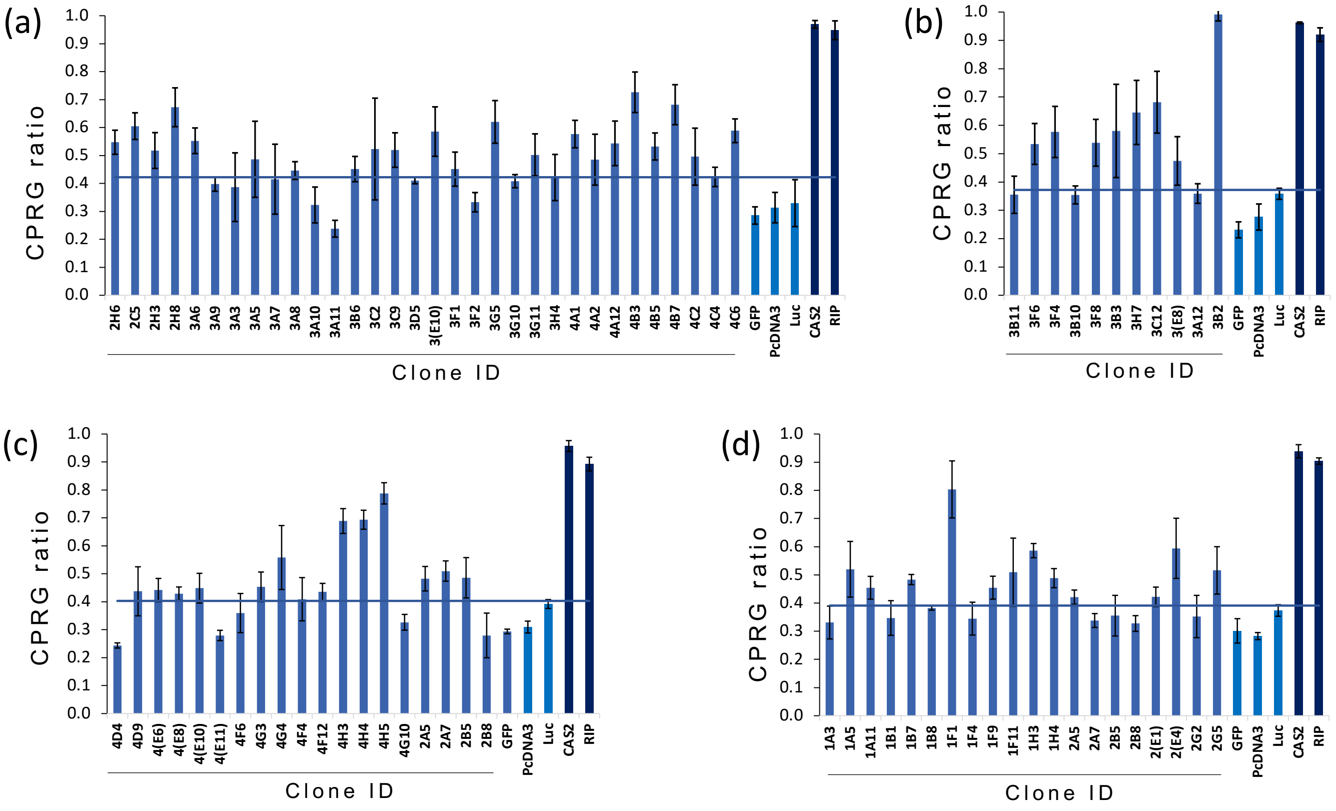

Supplementary Figure S3: Round-4 of genetic screen for anticancer genes. Plasmid DNA of 78 gene candidates selected from the third round of screening were purified using standard miniprep and co-transfected with a β-Gal-expressing plasmid in the CV-1 cells in four 96-well plates (a), (b), (c) and (d) – one gene per well. In all cases, *GFP*, luciferase (*Luc*) and pcDNA3 were used as negative controls whilst *CAS2* and *RIP* were used as positive controls. The horizonal line drawn at the upper range of negative control was used as selection threshold. Genes with CPRG ratios equal or below threshold were selected for the subsequent screening. Histograms represent the average ± SD of 3 independent transfections.

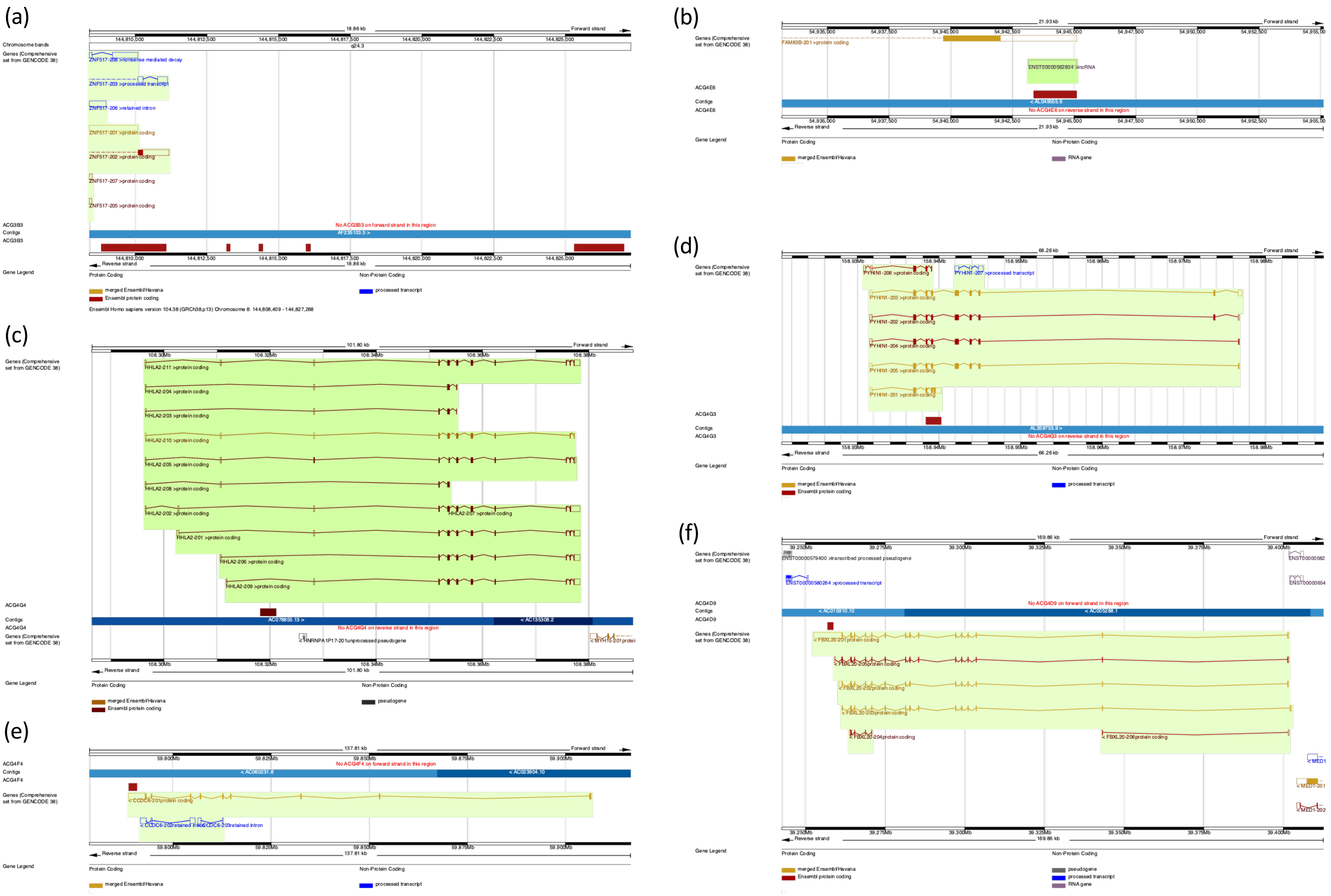

Supplementary Figure S4: Ensembl BLAT analysis of novel anticancer genes. (a) *ACG3B3* cDNA sequence exhibited typical exon structure but did not overlap any annotated gene on GENCODE 38 comprehensive database. (b) *ACG4E6* cDNA sequence overlapped to ENST00000562834 lncRNA, a novel transcript overlapping to *FAM83B* (c) *ACG4G4* cDNA sequence overlapped to intronic region of *HHLA2*. (d) *ACG4G3* cDNA sequence partially overlapped to 2-3 exons and intronic regions of *PYHIN1*. (e) *ACG4F4* cDNA sequence partially overlapped to last exon of *CCDC6*-201. (f) *ACG4D9* cDNA overlapped to last exon of *FBXL20*-201. Source: Ensembl *Homo sapiens* version 104.38 - http://ensembl.org/index.html.

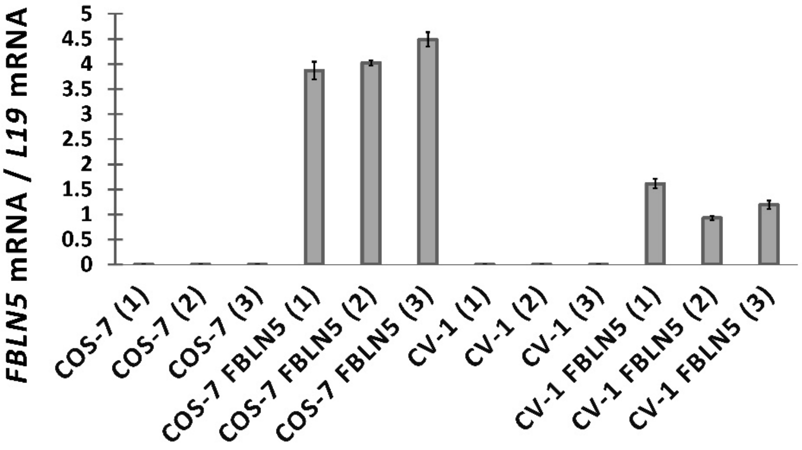

Supplementary Figure S5: Confirmation of FBLN5 overexpression in triplicate samples for transcriptomic analysis. COS-7 and CV-1 cells were transfected with a FBLN5-expressing plasmid and harvested 24 hours (COS-7) or 30 hours post-transfection (CV-1). RNA for each sample was extracted after pooling the cell lysates from 12 wells of a 24-well plate, each with an independently transfected population of cells. RNA for the untransfected CV-1 and COS-7 cells was also extracted by pooling lysates from 12 wells of 24-well plates for each sample. mRNA levels were calculated by RT-QPCR using relative standard curve method. *L19* was used as the normalizer gene. Data represent the average ± SD from 3 PCR-reactions.

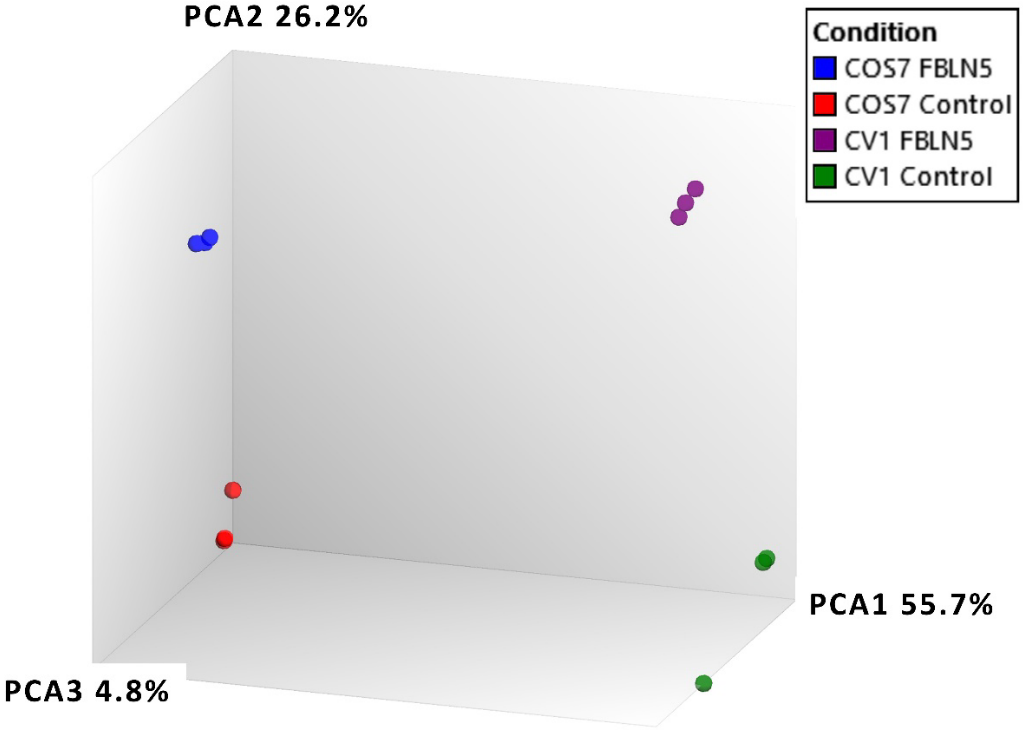

Supplementary Figure S6: Variance based principal component analysis depicting all 12 samples used in the transcriptomic analysis of wild type and *FBLN5*-transfected CV-1 and COS-7 cells. The biological replicates of CV-1 cells, COS-7 cells, FBLN5-transfected CV-1 cells and FBLN5-transfected-COS-7 cells exhibit distinct transcriptomic expression profiles. Results generated by Transcriptome Analysis Console (TAC) 4.0.1.

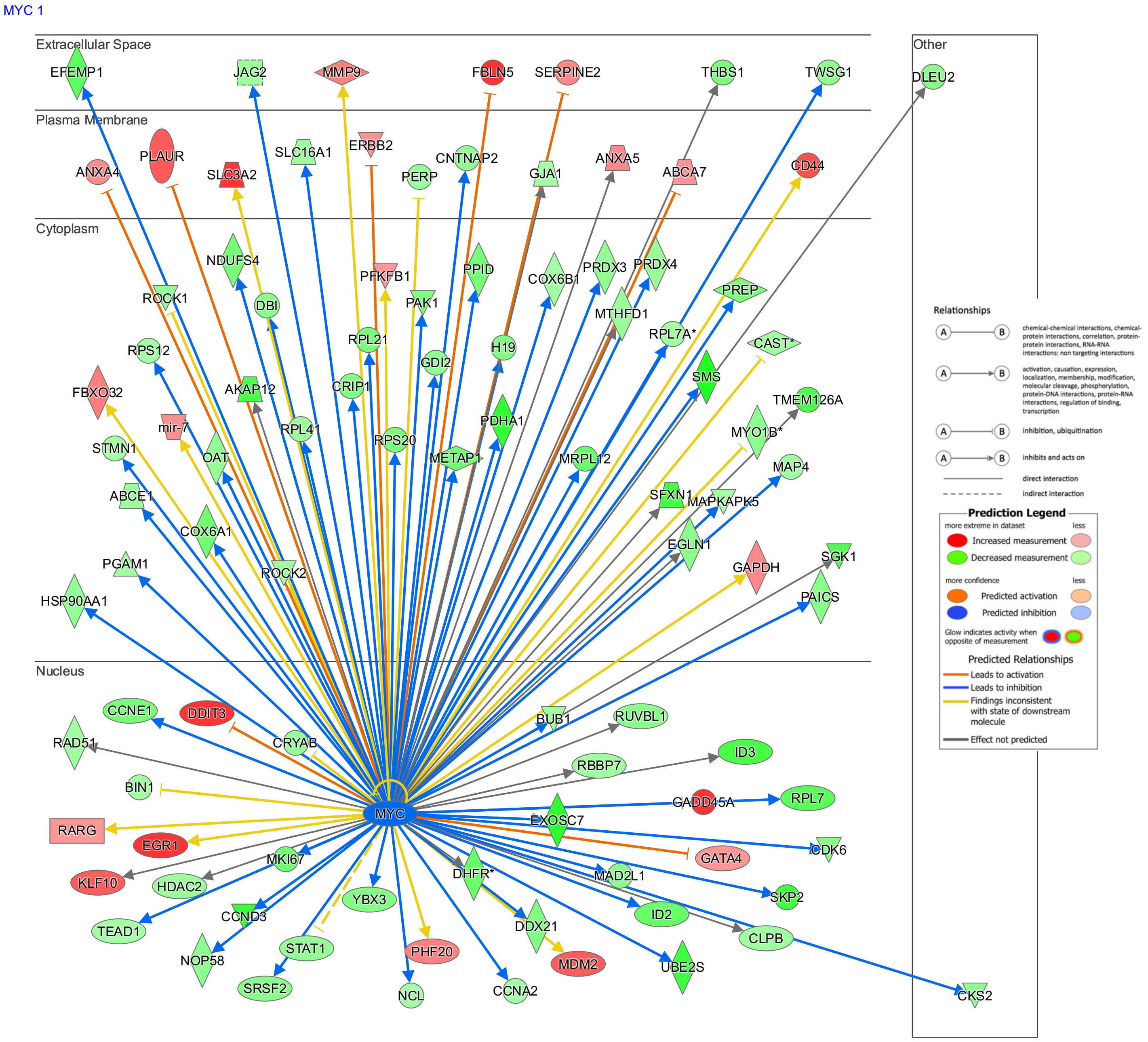

© 2000-2020 QIAGEN. All rights reserved.

Supplementary Figure S7: Network view of MYC as the predicted upstream regulator for the transcriptomic changes observed upon FBLN5 overexpression in COS-7 cells. IPA Upstream Regulator Analysis was run on the differentially expressed gene set between COS-7 cells transfected with *FBLN5* vs wild type COS-7 cells. A differential gene expression analysis filter comprising fold change ≥ 2 and ≤ -2, p ≤ 0.05 and FDR ≤ 0.05 was applied.

MYC 2

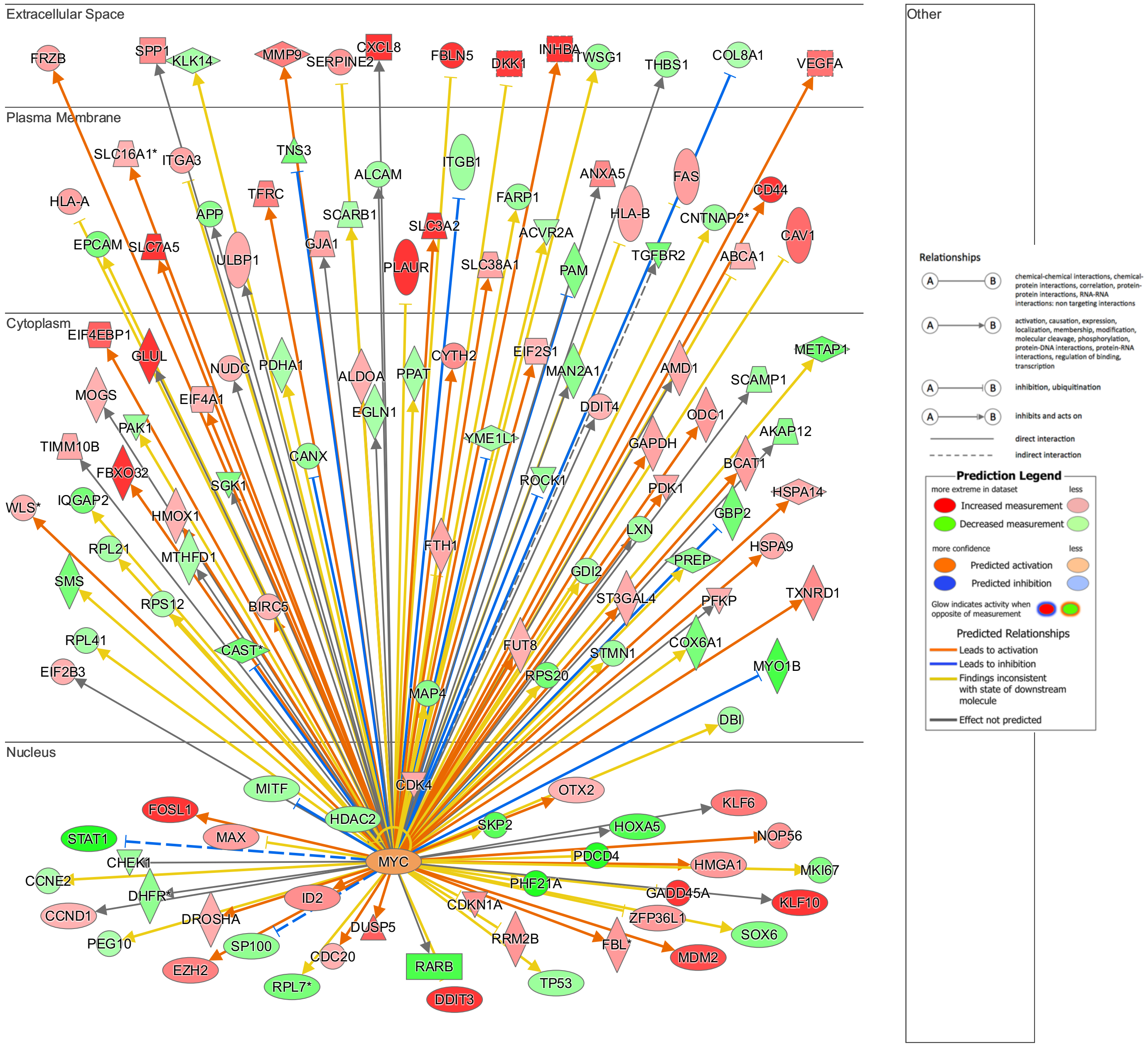

© 2000-2020 QIAGEN. All rights reserved.

Supplementary Figure S8: Network view of MYC as the predicted upstream regulator for the transcriptomic changes observed upon FBLN5 overexpression in CV-1 cells. IPA Upstream Regulator Analysis was run on the differentially expressed gene set between CV-1 cells transfected with *FBLN5* vs wild type CV-1 cells. A differential gene expression analysis filter comprising fold change ≥ 2 and ≤ -2, p ≤ 0.05 and FDR ≤ 0.05 was applied.

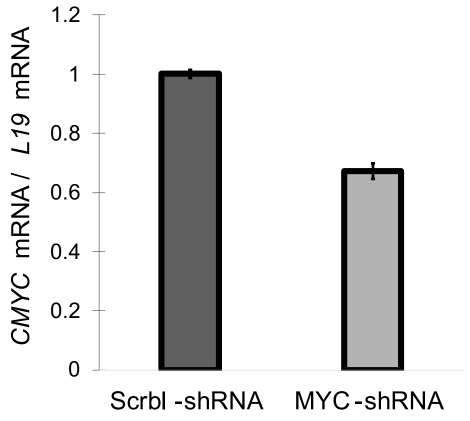

Supplementary Figure S9: Confirmation of stable *MYC* knockdown in MCF-7 cells. RT-QPCR analysis of MCF-7 control cells (Scrbl-shRNA) and MYC shRNA stable MCF-7 cells (MYC-shRNA) using the relative standard curve method. *L19* was used as the normalizer gene. Data show the average ± SD from two technical replicates.

Supplementary Movie M1: COS_7_FBLN5.avi, Time lapse series micrographs of COS-7 cells transfected with GFP and FBLN5 in 1:4 ratio. GFP expression in log_{10} scale.

Supplementary Movie M2: COS_7_LUC.avi, Time lapse series micrographs of COS-7 cells transfected with GFP and luciferase (negative control) in 1:4 ratio. GFP expression in log_{10} scale.

Supplementary Movie M3: COS_7_tBID.avi, Time lapse series micrographs of COS-7 cells transfected with GFP and tBID (positive control) in 1:4 ratio. GFP expression in log_{10} scale.

Supplementary Table TS1: $-\log_{10}$(Benjimini-Hochberg p-value) and z-score for the upstream regulator comparison analysis with 5 highest $-\log_{10}$(p-values) in COS-7 *vs* CV-1 dataset.

**Upstream Regulators**	**COS-7 vs CV-1**		**COS-7 FBLN5**		**CV-1 FBLN5**	
	**-log(p value)**	**Activation z-score**	**-log(p value)**	**Activation z-score**	**-log(p value)**	**Activation z-score**
ERBB2	20.31	4.31	1.58	-0.35	6.62	2.95
MYC	20.04	6.41	3.01	-4.65	5.27	0.93
KRAS	19.14	0.63	5.00	2.54	12.14	2.93
TP53	18.38	-2.98	4.04	0.21	6.19	0.21
E2F1	18.09	4.21	3.71	-3.35	2.95	-1.85

Comparison analysis results are based on the IPA Upstream Regulator analyses for differentially expressed genes between 'wild type COS-7 cells *vs* wild type CV-1 cells', 'COS-7 cells transiently transfected with *FBLN5* *vs* wild type COS-7 cells' and 'CV-1 cells transiently transfected with FBLN5 *vs* wild type CV-1 cells'.
